# *catena*-Poly[[(tetra­hydro­furan-κ*O*)lithium(I)]-bis­(μ-trimethyl­silanolato-κ^2^ *O*:*O*)-gallium(III)-bis­(μ-trimethyl­silanolato-κ^2^ *O*:*O*)-[(tetra­hydro­furan-κ*O*)lithium(I)]-μ-bromido]

**DOI:** 10.1107/S1600536810035518

**Published:** 2010-09-11

**Authors:** Rafał Grubba, Katarzyna Baranowska, Jerzy Pikies

**Affiliations:** a Department of Inorganic Chemistry, Faculty of Chemistry, Gdańsk University of Technology, 11/12 G. Narutowicz St., 80233 - PL Gdańsk, Poland

## Abstract

The title chain polymer compound, [GaLi_2_Br(C_3_H_9_OSi)_4_(C_4_H_8_O)_2_]_*n*_, was obtained in the reaction of GaBr_3_ with Me_3_SiOLi in toluene/tetra­hydro­furan. The Ga^III^ atom, located on a twofold rotation axis, is coordinated by four trimethyl­silanolate ligands and has a distorted tetra­hedral geometry. The Li^I^ atom is four coordinated by one bridging Br atom located on an inversion centre, two trimethyl­silanolate ligands and one tetra­hydro­furane mol­ecule in a distorted tetra­hedral geometry. The polymeric chains extend along [001]. The tetra­hydro­furane mol­ecule is disordered over two positions with site-occupancy factors of 0.57 (2) and 0.43 (2).

## Related literature

For the structures of similar compounds, see: Wheatley (1963 ▶); Barry & Richeson (1994 ▶); Chisholm *et al.* (2001 ▶). For the properties of GaBr, see: Dohmeier *et al.* (1996 ▶).
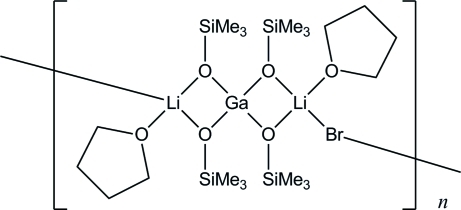


## Experimental

### 

#### Crystal data

[GaLi_2_Br(C_3_H_9_OSi)_4_(C_4_H_8_O)_2_]
*M* *_r_* = 664.49
Monoclinic, 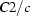

*a* = 25.802 (8) Å
*b* = 9.761 (2) Å
*c* = 18.689 (6) Å
β = 130.81 (2)°
*V* = 3563 (2) Å^3^
*Z* = 4
Mo *K*α radiation
μ = 2.06 mm^−1^
*T* = 150 K
0.2 × 0.18 × 0.09 mm

#### Data collection

Stoe Stadi IPDS 2 diffractometer
Absorption correction: numerical (*X-RED32*; Stoe & Cie, 2008 ▶) *T*_min_ = 0.503, *T*_max_ = 0.734
19442 measured reflections
3098 independent reflections
2926 reflections with *I* > 2σ(*I*)
*R*_int_ = 0.080

#### Refinement

*R*[*F* ^2^ > 2σ(*F* ^2^)] = 0.031
*wR*(*F* ^2^) = 0.084
*S* = 1.05
3098 reflections
199 parameters
H-atom parameters constrained
Δρ_max_ = 0.44 e Å^−3^
Δρ_min_ = −0.60 e Å^−3^

### 

Data collection: *IPDS* (Stoe & Cie, 2008 ▶); cell refinement: *IPDS*; data reduction: *X-RED32* (Stoe & Cie, 2008 ▶); program(s) used to solve structure: *SHELXS97* (Sheldrick, 2008 ▶); program(s) used to refine structure: *SHELXL97* (Sheldrick, 2008 ▶); molecular graphics: *ORTEP-3 for Windows* (Farrugia, 1997 ▶); software used to prepare material for publication: *WinGX* (Farrugia, 1999 ▶).

## Supplementary Material

Crystal structure: contains datablocks I, global. DOI: 10.1107/S1600536810035518/is2596sup1.cif

Structure factors: contains datablocks I. DOI: 10.1107/S1600536810035518/is2596Isup2.hkl

Additional supplementary materials:  crystallographic information; 3D view; checkCIF report

## Figures and Tables

**Fig. 1.**
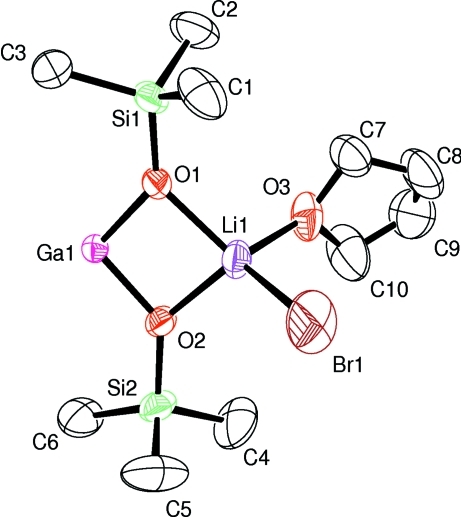
The molecular structure of the asymmetric unit of (I), with the atomic numbering scheme. Displacement ellipsoids are drawn at the 50% probability level. H atoms have been omitted.

**Fig. 2.**
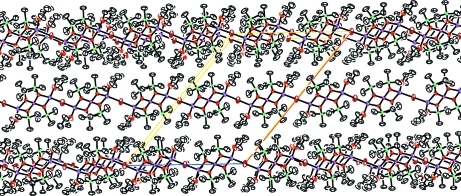
The polymeric chains formed by (I) in the crystal. H atoms have been omitted.

## supplementary crystallographic information

### Comment

Complex (I) was synthesized in the course of our studies on gallium clusters. The compound was obtained in the reaction of GaBr with Me_3_SiOLi. GaBr is unstable in solution and easily disproportionates to the metallic gallium, gallium clusters, and GaBr_3 _(Dohmeier *et al.*, 1996). The direct reaction of GaBr_3_ with Me_3_SiOLi leads to complex (I).

The polymeric structure of (I) consist of {[Li(THF)][LiBr(THF)][Ga(OSiMe_3_)_4_]} moieties with gallium atom situated in the central position. The molecular structure of the monomeric unit of (I) is shown in Fig. 1. The central gallium has a distorted tetrahedral geometry and is coordinated by four O atoms from trimethylsilanolate ligands. Each OSiMe_3_ ligand is a bridging one and additionally coordinates to the lithium atom. The Ga atom, two O atoms of siloxo ligands and Li atoms form a distorted planar square. Each Li atom is four coordinated by one Br atom, two O atoms from OSiMe_3_ ligands and one O atom from a molecule of tetrahydrofurane in a distorted tetrahedral geometry. To the best of our knowledge, only three examples of gallium complexes with OSiMe_3_ ligands are known (Wheatley, 1963; Barry & Richeson, 1994; Chisholm *et al.*, 2001). [Li(THF)_2_[Ga(N(SiMe_3_)_2_(OSiMe_3_)_2_Cl] obtained by Barry & Richeson is very similar to complex (I). Both complexes contain distorted planar square formed by Ga1—O1—Li—O2 with comparable Ga—O distances: [1.848 (9) and 1.872 (1) Å (Barry & Richeson, 1994); 1.8207 (13) and 1.818 (14) Å (I)] and Li—O distances [1.90 (3) and 1.98 (3) Å (Barry & Richeson, 1994); 2.011 (4) and 1.972 (4) Å (I)].

### Experimental

Solution of GaBr (6,40 mmol) in toluene/THF (20 ml, 3:1) (Dohmeier *et al.*, 1996) was added dropwise to solution of Me_3_SiOLi (0,813 g, 8,47 mmol) in THF (20 ml) at -78 °C. Afterwards, there action mixture was stirred at room temperature overnight. The solution was filtred and concentrated *in vacuo* to half volume. Within a few days, orange crystals of (I) were formed solution at room temperature.

### Refinement

Hydrogen atoms were placed in geometrically calculated positions (C—H 0.98 Å for methyl and 0.99 Å for methylene H atoms) and refined as riding on their parent atoms, with *U*_iso_(H) = 1.2*U*_eq_(C) for methylene and 1.5*U*_eq_(C) for methyl groups. Atoms C7—C10 of one THF are disordered over two positions with site occupancy factors of 0.57 (2) and 0.43 (2).

### Crystal data

**Table:**

[GaLi_2_Br(C_3_H_9_OSi)_4_(C_4_H_8_O)_2_]	*F*(000) = 1392
*M**_r_* = 664.49	*D*_x_ = 1.239 Mg m^−^^3^
Monoclinic, *C*2/*c*	Mo *K*α radiation, λ = 0.71073 Å
Hall symbol: -C 2yc	Cell parameters from 29109 reflections
*a* = 25.802 (8) Å	θ = 1.4–25.0°
*b* = 9.761 (2) Å	µ = 2.06 mm^−^^1^
*c* = 18.689 (6) Å	*T* = 150 K
β = 130.81 (2)°	Prism, colourless
*V* = 3563 (2) Å^3^	0.2 × 0.18 × 0.09 mm
*Z* = 4	

### Data collection

**Table:**

Stoe Stadi IPDS 2 diffractometer	3098 independent reflections
Radiation source: fine-focus sealed tube	2926 reflections with *I* > 2σ(*I*)
graphite	*R*_int_ = 0.080
Detector resolution: 6.67 pixels mm^-1^	θ_max_ = 25.0°, θ_min_ = 2.2°
ω scan	*h* = −30→30
Absorption correction: numerical (*X-RED32*; Stoe & Cie, 2008)	*k* = −11→11
*T*_min_ = 0.503, *T*_max_ = 0.734	*l* = −22→22
19442 measured reflections	

### Refinement

**Table:**

Refinement on *F*^2^	Primary atom site location: structure-invariant direct methods
Least-squares matrix: full	Secondary atom site location: difference Fourier map
*R*[*F*^2^ > 2σ(*F*^2^)] = 0.031	Hydrogen site location: inferred from neighbouring sites
*wR*(*F*^2^) = 0.084	H-atom parameters constrained
*S* = 1.05	*w* = 1/[σ^2^(*F*_o_^2^) + (0.0478*P*)^2^ + 2.5155*P*] where *P* = (*F*_o_^2^ + 2*F*_c_^2^)/3
3098 reflections	(Δ/σ)_max_ < 0.001
199 parameters	Δρ_max_ = 0.44 e Å^−^^3^
0 restraints	Δρ_min_ = −0.59 e Å^−^^3^

### Special details

**Table:**

Geometry. All s.u.'s (except the s.u. in the dihedral angle between two l.s. planes) are estimated using the full covariance matrix. The cell s.u.'s are taken into account individually in the estimation of s.u.'s in distances, angles and torsion angles; correlations between s.u.'s in cell parameters are only used when they are defined by crystal symmetry. An approximate (isotropic) treatment of cell s.u.'s is used for estimating s.u.'s involving l.s. planes.
Refinement. Refinement of *F*^2^ against ALL reflections. The weighted *R*-factor *wR* and goodness of fit *S* are based on *F*^2^, conventional *R*-factors *R* are based on *F*, with *F* set to zero for negative *F*^2^. The threshold expression of *F*^2^ > 2σ(*F*^2^) is used only for calculating *R*-factors(gt) *etc*. and is not relevant to the choice of reflections for refinement. *R*-factors based on *F*^2^ are statistically about twice as large as those based on *F*, and *R*- factors based on ALL data will be even larger.

### Fractional atomic coordinates and isotropic or equivalent isotropic displacement parameters (Å^2^)

**Table:**

	*x*	*y*	*z*	*U*_iso_*/*U*_eq_	Occ. (<1)
Br1	0	0	0	0.1153 (3)	
Ga1	0	0.08220 (2)	0.25	0.02639 (11)	
Si1	0.11720 (3)	−0.13978 (6)	0.32580 (5)	0.04353 (16)	
Si2	−0.08944 (3)	0.28619 (7)	0.07510 (4)	0.04904 (18)	
O1	0.06525 (7)	−0.01375 (13)	0.26059 (9)	0.0343 (3)	
O2	−0.02655 (7)	0.18106 (14)	0.14841 (9)	0.0366 (3)	
O3	0.12462 (10)	0.19275 (18)	0.19590 (15)	0.0616 (5)	
C1	0.11245 (16)	−0.2675 (3)	0.2482 (3)	0.0730 (8)	
H1A	0.124	−0.2233	0.2131	0.109*	
H1B	0.0659	−0.3049	0.2036	0.109*	
H1C	0.1449	−0.3419	0.2869	0.109*	
C2	0.20664 (14)	−0.0753 (4)	0.4125 (2)	0.0775 (9)	
H2A	0.2116	−0.0186	0.4599	0.116*	
H2B	0.2171	−0.0203	0.3795	0.116*	
H2C	0.2383	−0.153	0.4437	0.116*	
C3	0.09372 (16)	−0.2196 (3)	0.3913 (2)	0.0759 (9)	
H3A	0.0489	−0.2639	0.3465	0.114*	
H3B	0.0917	−0.149	0.4267	0.114*	
H3C	0.1282	−0.2883	0.4354	0.114*	
C5	−0.15978 (19)	0.1939 (5)	−0.0340 (2)	0.1035 (14)	
H5A	−0.1746	0.1167	−0.0177	0.155*	
H5B	−0.1436	0.1598	−0.0657	0.155*	
H5C	−0.1984	0.2565	−0.0763	0.155*	
C4	−0.0577 (2)	0.4234 (4)	0.0441 (3)	0.0990 (13)	
H4A	−0.0466	0.3846	0.0072	0.149*	
H4B	−0.0165	0.4648	0.1021	0.149*	
H4C	−0.0932	0.4937	0.0066	0.149*	
C6	−0.12074 (18)	0.3621 (3)	0.1317 (2)	0.0776 (9)	
H6A	−0.0833	0.4119	0.1887	0.116*	
H6B	−0.137	0.289	0.1487	0.116*	
H6C	−0.1584	0.4254	0.0874	0.116*	
Li1	0.0429 (2)	0.0908 (4)	0.1507 (3)	0.0437 (8)	
C7	0.1816 (6)	0.1116 (11)	0.2192 (8)	0.064 (2)	0.57 (2)
H7A	0.2195	0.108	0.2885	0.077*	0.57 (2)
H7B	0.1665	0.0169	0.1951	0.077*	0.57 (2)
C8	0.2033 (6)	0.1793 (15)	0.1742 (11)	0.092 (4)	0.57 (2)
H8A	0.2529	0.1655	0.2106	0.11*	0.57 (2)
H8B	0.1776	0.1448	0.1091	0.11*	0.57 (2)
C9	0.1886 (8)	0.3183 (16)	0.1736 (15)	0.102 (5)	0.57 (2)
H9A	0.1799	0.3672	0.1203	0.123*	0.57 (2)
H9B	0.2271	0.3635	0.2335	0.123*	0.57 (2)
C10	0.1277 (9)	0.3169 (18)	0.1630 (13)	0.102 (5)	0.57 (2)
H10A	0.1295	0.3931	0.1995	0.123*	0.57 (2)
H10B	0.0864	0.3289	0.0956	0.123*	0.57 (2)
C7A	0.1685 (12)	0.126 (2)	0.187 (2)	0.125 (8)	0.43 (2)
H7C	0.1421	0.0747	0.1268	0.15*	0.43 (2)
H7D	0.1992	0.0611	0.2403	0.15*	0.43 (2)
C8A	0.2101 (9)	0.247 (4)	0.1900 (10)	0.146 (15)	0.43 (2)
H8C	0.2596	0.2374	0.2454	0.175*	0.43 (2)
H8D	0.204	0.2473	0.1319	0.175*	0.43 (2)
C9A	0.1826 (8)	0.375 (2)	0.1970 (14)	0.110 (8)	0.43 (2)
H9C	0.1813	0.4493	0.1602	0.132*	0.43 (2)
H9D	0.2106	0.4041	0.2637	0.132*	0.43 (2)
C10A	0.1110 (9)	0.336 (2)	0.1559 (9)	0.076 (4)	0.43 (2)
H10C	0.0937	0.3971	0.1785	0.091*	0.43 (2)
H10D	0.0784	0.3355	0.086	0.091*	0.43 (2)

### Atomic displacement parameters (Å^2^)

**Table:**

	*U*^11^	*U*^22^	*U*^33^	*U*^12^	*U*^13^	*U*^23^
Br1	0.1081 (4)	0.1890 (7)	0.0632 (3)	−0.0066 (4)	0.0623 (3)	−0.0435 (4)
Ga1	0.02674 (16)	0.02626 (16)	0.02663 (16)	0	0.01764 (13)	0
Si1	0.0344 (3)	0.0407 (3)	0.0561 (4)	0.0103 (2)	0.0299 (3)	0.0130 (3)
Si2	0.0548 (4)	0.0490 (3)	0.0400 (3)	0.0205 (3)	0.0295 (3)	0.0161 (3)
O1	0.0327 (7)	0.0349 (6)	0.0390 (7)	0.0039 (5)	0.0251 (6)	0.0045 (5)
O2	0.0420 (7)	0.0371 (7)	0.0338 (7)	0.0091 (6)	0.0261 (6)	0.0076 (5)
O3	0.0746 (12)	0.0558 (9)	0.0834 (13)	−0.0096 (9)	0.0644 (11)	0.0009 (9)
C1	0.0711 (18)	0.0480 (13)	0.115 (2)	0.0111 (13)	0.0675 (19)	−0.0024 (15)
C2	0.0367 (13)	0.092 (2)	0.075 (2)	0.0094 (13)	0.0240 (14)	0.0147 (16)
C3	0.0673 (17)	0.0797 (19)	0.091 (2)	0.0280 (15)	0.0563 (18)	0.0480 (17)
C5	0.072 (2)	0.118 (3)	0.0516 (17)	0.026 (2)	0.0097 (16)	−0.0016 (18)
C4	0.137 (4)	0.076 (2)	0.121 (3)	0.044 (2)	0.101 (3)	0.057 (2)
C6	0.082 (2)	0.082 (2)	0.0751 (19)	0.0426 (17)	0.0540 (18)	0.0226 (16)
Li1	0.051 (2)	0.0485 (19)	0.0458 (19)	0.0031 (15)	0.0378 (18)	0.0035 (15)
C7	0.046 (3)	0.068 (4)	0.076 (4)	−0.003 (3)	0.038 (3)	0.002 (3)
C8	0.056 (5)	0.108 (8)	0.126 (9)	−0.001 (5)	0.066 (6)	0.017 (6)
C9	0.088 (8)	0.102 (11)	0.123 (12)	−0.003 (7)	0.072 (9)	0.038 (8)
C10	0.112 (10)	0.063 (5)	0.182 (13)	0.018 (6)	0.118 (11)	0.042 (6)
C7A	0.084 (12)	0.167 (14)	0.17 (2)	0.014 (10)	0.103 (14)	0.016 (13)
C8A	0.077 (10)	0.31 (4)	0.058 (6)	−0.118 (19)	0.047 (6)	−0.039 (14)
C9A	0.063 (8)	0.116 (13)	0.079 (8)	−0.044 (8)	0.015 (6)	0.038 (8)
C10A	0.059 (7)	0.079 (10)	0.060 (6)	−0.020 (6)	0.027 (5)	0.019 (5)

### Geometric parameters (Å, °)

**Table:**

Br1—Li1	2.422 (4)	C5—H5A	0.98
Br1—Li1^i^	2.422 (4)	C5—H5B	0.98
Ga1—O2^ii^	1.8180 (14)	C5—H5C	0.98
Ga1—O2	1.8180 (14)	C4—H4A	0.98
Ga1—O1^ii^	1.8207 (13)	C4—H4B	0.98
Ga1—O1	1.8207 (13)	C4—H4C	0.98
Ga1—Li1^ii^	2.713 (3)	C6—H6A	0.98
Ga1—Li1	2.713 (3)	C6—H6B	0.98
Si1—O1	1.6310 (14)	C6—H6C	0.98
Si1—C1	1.855 (3)	C7—C8	1.441 (18)
Si1—C3	1.855 (3)	C7—H7A	0.99
Si1—C2	1.860 (3)	C7—H7B	0.99
Si2—O2	1.6301 (14)	C8—C9	1.41 (2)
Si2—C4	1.850 (3)	C8—H8A	0.99
Si2—C5	1.851 (4)	C8—H8B	0.99
Si2—C6	1.853 (3)	C9—C10	1.45 (2)
O1—Li1	2.011 (4)	C9—H9A	0.99
O2—Li1	1.972 (4)	C9—H9B	0.99
O3—C10	1.385 (17)	C10—H10A	0.99
O3—C7A	1.41 (2)	C10—H10B	0.99
O3—C7	1.463 (14)	C7A—C8A	1.58 (3)
O3—C10A	1.513 (19)	C7A—H7C	0.99
O3—Li1	1.955 (4)	C7A—H7D	0.99
C1—H1A	0.98	C8A—C9A	1.48 (3)
C1—H1B	0.98	C8A—H8C	0.99
C1—H1C	0.98	C8A—H8D	0.99
C2—H2A	0.98	C9A—C10A	1.51 (2)
C2—H2B	0.98	C9A—H9C	0.99
C2—H2C	0.98	C9A—H9D	0.99
C3—H3A	0.98	C10A—H10C	0.99
C3—H3B	0.98	C10A—H10D	0.99
C3—H3C	0.98		
			
Li1—Br1—Li1^i^	180.00 (12)	H4A—C4—H4B	109.5
O2^ii^—Ga1—O2	115.88 (9)	Si2—C4—H4C	109.5
O2^ii^—Ga1—O1^ii^	94.42 (6)	H4A—C4—H4C	109.5
O2—Ga1—O1^ii^	117.97 (7)	H4B—C4—H4C	109.5
O2^ii^—Ga1—O1	117.97 (7)	Si2—C6—H6A	109.5
O2—Ga1—O1	94.42 (6)	Si2—C6—H6B	109.5
O1^ii^—Ga1—O1	118.09 (8)	H6A—C6—H6B	109.5
O2^ii^—Ga1—Li1^ii^	46.60 (9)	Si2—C6—H6C	109.5
O2—Ga1—Li1^ii^	130.87 (9)	H6A—C6—H6C	109.5
O1^ii^—Ga1—Li1^ii^	47.83 (9)	H6B—C6—H6C	109.5
O1—Ga1—Li1^ii^	134.67 (9)	O3—Li1—O2	119.32 (19)
O2^ii^—Ga1—Li1	130.87 (9)	O3—Li1—O1	108.0 (2)
O2—Ga1—Li1	46.60 (9)	O2—Li1—O1	84.19 (14)
O1^ii^—Ga1—Li1	134.67 (9)	O3—Li1—Br1	103.47 (15)
O1—Ga1—Li1	47.83 (9)	O2—Li1—Br1	114.85 (18)
Li1^ii^—Ga1—Li1	176.47 (15)	O1—Li1—Br1	127.60 (17)
O1—Si1—C1	108.71 (12)	O3—Li1—Ga1	121.79 (17)
O1—Si1—C3	110.29 (10)	O2—Li1—Ga1	42.05 (7)
C1—Si1—C3	110.49 (15)	O1—Li1—Ga1	42.14 (7)
O1—Si1—C2	109.97 (12)	Br1—Li1—Ga1	134.71 (16)
C1—Si1—C2	108.59 (15)	C8—C7—O3	106.1 (8)
C3—Si1—C2	108.77 (16)	C8—C7—H7A	110.5
O2—Si2—C4	108.53 (14)	O3—C7—H7A	110.5
O2—Si2—C5	109.90 (14)	C8—C7—H7B	110.5
C4—Si2—C5	108.9 (2)	O3—C7—H7B	110.5
O2—Si2—C6	109.74 (11)	H7A—C7—H7B	108.7
C4—Si2—C6	109.66 (17)	C9—C8—C7	104.0 (12)
C5—Si2—C6	110.09 (18)	C9—C8—H8A	111
Si1—O1—Ga1	135.98 (8)	C7—C8—H8A	111
Si1—O1—Li1	133.87 (13)	C9—C8—H8B	111
Ga1—O1—Li1	90.02 (11)	C7—C8—H8B	111
Si2—O2—Ga1	134.48 (8)	H8A—C8—H8B	109
Si2—O2—Li1	133.68 (12)	C8—C9—C10	104.8 (12)
Ga1—O2—Li1	91.34 (11)	C8—C9—H9A	110.8
C10—O3—C7A	94.2 (11)	C10—C9—H9A	110.8
C10—O3—C7	105.5 (8)	C8—C9—H9B	110.8
C7A—O3—C10A	108.6 (12)	C10—C9—H9B	110.8
C7—O3—C10A	120.7 (10)	H9A—C9—H9B	108.9
C10—O3—Li1	127.8 (7)	O3—C10—C9	108.6 (12)
C7A—O3—Li1	115.8 (9)	O3—C10—H10A	110
C7—O3—Li1	116.3 (4)	C9—C10—H10A	110
C10A—O3—Li1	114.8 (7)	O3—C10—H10B	110
Si1—C1—H1A	109.5	C9—C10—H10B	110
Si1—C1—H1B	109.5	H10A—C10—H10B	108.3
H1A—C1—H1B	109.5	O3—C7A—C8A	103.3 (18)
Si1—C1—H1C	109.5	O3—C7A—H7C	111.1
H1A—C1—H1C	109.5	C8A—C7A—H7C	111.1
H1B—C1—H1C	109.5	O3—C7A—H7D	111.1
Si1—C2—H2A	109.5	C8A—C7A—H7D	111.1
Si1—C2—H2B	109.5	H7C—C7A—H7D	109.1
H2A—C2—H2B	109.5	C9A—C8A—C7A	106.1 (14)
Si1—C2—H2C	109.5	C9A—C8A—H8C	110.5
H2A—C2—H2C	109.5	C7A—C8A—H8C	110.5
H2B—C2—H2C	109.5	C9A—C8A—H8D	110.5
Si1—C3—H3A	109.5	C7A—C8A—H8D	110.5
Si1—C3—H3B	109.5	H8C—C8A—H8D	108.7
H3A—C3—H3B	109.5	C8A—C9A—C10A	104.0 (13)
Si1—C3—H3C	109.5	C8A—C9A—H9C	111
H3A—C3—H3C	109.5	C10A—C9A—H9C	111
H3B—C3—H3C	109.5	C8A—C9A—H9D	111
Si2—C5—H5A	109.5	C10A—C9A—H9D	111
Si2—C5—H5B	109.5	H9C—C9A—H9D	109
H5A—C5—H5B	109.5	O3—C10A—C9A	99.6 (12)
Si2—C5—H5C	109.5	O3—C10A—H10C	111.9
H5A—C5—H5C	109.5	C9A—C10A—H10C	111.9
H5B—C5—H5C	109.5	O3—C10A—H10D	111.9
Si2—C4—H4A	109.5	C9A—C10A—H10D	111.9
Si2—C4—H4B	109.5	H10C—C10A—H10D	109.6
			
C1—Si1—O1—Ga1	132.69 (14)	Si2—O2—Li1—O1	−171.36 (12)
C3—Si1—O1—Ga1	11.41 (19)	Ga1—O2—Li1—O1	1.13 (11)
C2—Si1—O1—Ga1	−108.54 (17)	Si2—O2—Li1—Br1	−42.4 (3)
C1—Si1—O1—Li1	−41.9 (2)	Ga1—O2—Li1—Br1	130.09 (14)
C3—Si1—O1—Li1	−163.2 (2)	Si2—O2—Li1—Ga1	−172.49 (19)
C2—Si1—O1—Li1	76.8 (2)	Si1—O1—Li1—O3	−65.9 (2)
O2^ii^—Ga1—O1—Si1	62.64 (14)	Ga1—O1—Li1—O3	117.85 (16)
O2—Ga1—O1—Si1	−174.90 (12)	Si1—O1—Li1—O2	175.13 (12)
O1^ii^—Ga1—O1—Si1	−49.84 (10)	Ga1—O1—Li1—O2	−1.13 (11)
Li1^ii^—Ga1—O1—Si1	7.31 (19)	Si1—O1—Li1—Br1	58.1 (3)
Li1—Ga1—O1—Si1	−176.12 (19)	Ga1—O1—Li1—Br1	−118.2 (2)
O2^ii^—Ga1—O1—Li1	−121.24 (13)	Si1—O1—Li1—Ga1	176.26 (18)
O2—Ga1—O1—Li1	1.22 (12)	O2^ii^—Ga1—Li1—O3	11.3 (3)
O1^ii^—Ga1—O1—Li1	126.28 (12)	O2—Ga1—Li1—O3	100.0 (2)
Li1^ii^—Ga1—O1—Li1	−176.57 (15)	O1^ii^—Ga1—Li1—O3	−171.58 (13)
C4—Si2—O2—Ga1	143.96 (18)	O1—Ga1—Li1—O3	−81.7 (2)
C5—Si2—O2—Ga1	−97.1 (2)	O2^ii^—Ga1—Li1—O2	−88.64 (16)
C6—Si2—O2—Ga1	24.14 (19)	O1^ii^—Ga1—Li1—O2	88.45 (14)
C4—Si2—O2—Li1	−46.6 (2)	O1—Ga1—Li1—O2	178.32 (17)
C5—Si2—O2—Li1	72.4 (2)	O2^ii^—Ga1—Li1—O1	93.04 (13)
C6—Si2—O2—Li1	−166.4 (2)	O2—Ga1—Li1—O1	−178.32 (17)
O2^ii^—Ga1—O2—Si2	−64.78 (11)	O1^ii^—Ga1—Li1—O1	−89.87 (16)
O1^ii^—Ga1—O2—Si2	45.99 (14)	O2^ii^—Ga1—Li1—Br1	−166.26 (14)
O1—Ga1—O2—Si2	171.14 (12)	O2—Ga1—Li1—Br1	−77.6 (2)
Li1^ii^—Ga1—O2—Si2	−10.94 (19)	O1^ii^—Ga1—Li1—Br1	10.8 (3)
Li1—Ga1—O2—Si2	172.39 (19)	O1—Ga1—Li1—Br1	100.7 (2)
O2^ii^—Ga1—O2—Li1	122.83 (13)	C10—O3—C7—C8	−15.5 (15)
O1^ii^—Ga1—O2—Li1	−126.40 (13)	C7A—O3—C7—C8	39 (3)
O1—Ga1—O2—Li1	−1.25 (13)	C10A—O3—C7—C8	−14.6 (13)
Li1^ii^—Ga1—O2—Li1	176.67 (14)	Li1—O3—C7—C8	132.3 (9)
C10—O3—Li1—O2	−56.0 (10)	O3—C7—C8—C9	30.4 (14)
C7A—O3—Li1—O2	−174.4 (14)	C7—C8—C9—C10	−33.0 (18)
C7—O3—Li1—O2	164.5 (6)	C7A—O3—C10—C9	−20 (2)
C10A—O3—Li1—O2	−46.6 (9)	C7—O3—C10—C9	−5.1 (18)
C10—O3—Li1—O1	−149.4 (10)	C10A—O3—C10—C9	178 (6)
C7A—O3—Li1—O1	92.1 (15)	Li1—O3—C10—C9	−147.9 (12)
C7—O3—Li1—O1	71.0 (6)	C8—C9—C10—O3	24 (2)
C10A—O3—Li1—O1	−140.1 (8)	C10—O3—C7A—C8A	22.8 (19)
C10—O3—Li1—Br1	73.1 (10)	C7—O3—C7A—C8A	−105 (4)
C7A—O3—Li1—Br1	−45.4 (15)	C10A—O3—C7A—C8A	28 (2)
C7—O3—Li1—Br1	−66.4 (6)	Li1—O3—C7A—C8A	158.7 (10)
C10A—O3—Li1—Br1	82.5 (8)	O3—C7A—C8A—C9A	−2(2)
C10—O3—Li1—Ga1	−105.1 (10)	C7A—C8A—C9A—C10A	−24.0 (16)
C7A—O3—Li1—Ga1	136.4 (14)	C10—O3—C10A—C9A	−23 (4)
C7—O3—Li1—Ga1	115.3 (6)	C7A—O3—C10A—C9A	−42.8 (19)
C10A—O3—Li1—Ga1	−95.8 (8)	C7—O3—C10A—C9A	−26.8 (14)
Si2—O2—Li1—O3	81.3 (3)	Li1—O3—C10A—C9A	−174.2 (11)
Ga1—O2—Li1—O3	−106.2 (2)	C8A—C9A—C10A—O3	39.0 (15)

Symmetry codes: (i) −*x*, −*y*, −*z*; (ii) −*x*, *y*, −*z*+1/2.
